# Nanoengineered Advanced Materials for Enabling Hydrogen Economy: Functionalized Graphene–Incorporated Cupric Oxide Catalyst for Efficient Solar Hydrogen Production

**DOI:** 10.1002/gch2.201900087

**Published:** 2020-01-24

**Authors:** Goutam Kumar Dalapati, Saeid Masudy‐Panah, Roozbeh Siavash Moakhar, Sabyasachi Chakrabortty, Siddhartha Ghosh, Ajay Kushwaha, Reza Katal, Chin Sheng Chua, Gong Xiao, Sudhiranjan Tripathy, Seeram Ramakrishna

**Affiliations:** ^1^ Department of Physics SRM University – AP Amaravati Andhra Pradesh 522502 India; ^2^ Institute of Materials Research and Engineering A*STAR (Agency for Science, Technology and Research) 2 Fusionopolis Way; Innovis, #08‐03 Singapore 138634 Singapore; ^3^ School of Engineering & Innovation The Open University Milton Keynes MK7 6AA UK; ^4^ Center for Nanofibers and Nanotechnology Faculty of Engineering National University of Singapore Singapore 117576 Singapore; ^5^ Energy Electronic Systems (LEES) Singapore‐MIT Alliance for Research and Technology (SMART) Centre 1 CREATE Way, #09‐01/02 CREATE Tower Singapore 138602 Singapore; ^6^ Electrical and Computer Engineering National University of Singapore Singapore 119260 Singapore; ^7^ Department of Materials Science and Engineering Sharif University of Technology Tehran 11155‐9466 Iran; ^8^ Department of Chemistry SRM University – AP Amaravati Andhra Pradesh 522502 India; ^9^ Discipline of Metallurgy Engineering and Materials Science Indian Institute of Technology Indore Simrol Indore Madhya Pradesh 453552 India; ^10^ Department of Civil & Environmental Engineering National University of Singapore Singapore 119260 Singapore

**Keywords:** photocatalytic degradation, photocorrosion stability, Raman spectroscopy, solar hydrogen

## Abstract

Cupric oxide (CuO) is a promising candidate as a photocathode for visible‐light‐driven photo‐electrochemical (PEC) water splitting. However, the stability of the CuO photocathode against photo‐corrosion is crucial for developing CuO‐based PEC cells. This study demonstrates a stable and efficient photocathode through the introduction of graphene into CuO film (CuO:G). The CuO:G composite electrodes are prepared using graphene‐incorporated CuO sol–gel solution via spin‐coating techniques. The graphene is modified with two different types of functional groups, such as amine (—NH_2_) and carboxylic acid (—COOH). The —COOH‐functionalized graphene incorporation into CuO photocathode exhibits better stability and also improves the photocurrent generation compare to control CuO electrode. In addition, —COOH‐functionalized graphene reduces the conversion of CuO phase into cuprous oxide (Cu_2_O) during photo‐electrochemical reaction due to effective charge transfer and leads to a more stable photocathode. The reduction of CuO to Cu_2_O phase is significantly lesser in CuO:G‐COOH as compared to CuO and CuO:G‐NH_2_ photocathodes. The photocatalytic degradation of methylene blue (MB) by CuO, CuO:G‐NH_2_ and CuO:G‐COOH is also investigated. By integrating CuO:G‐COOH photocathode with a sol–gel‐deposited TiO_2_ protecting layer and Au–Pd nanostructure, stable and efficient photocathode are developed for solar hydrogen generation.

## Graphical abstract

Graphene‐incorporated cupric oxide (CuO:G) is a stable and efficient photocathode. The CuO:G electrodes are prepared using sol–gel solution via spin‐coating. The –COOH‐functionalized graphene reduces CuO phase conversion into cuprous oxide (Cu_2_O) during photo‐electrochemical reaction and leads to a more stable photocathode for solar hydrogen generation. The CuO:G electrodes are also efficient for the dye degradation.

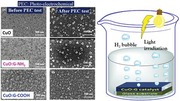

## 1 Introduction

Solar energy harvesting using photo‐electrochemical (PEC) water splitting is an emergent interest to convert solar energy into the fuel energy.^1^, ^2^, ^3^ The PEC water‐splitting technology requires semiconductor‐based photoelectrode to generate electron–hole pairs using solar light.^1^ Metal oxide‐based semiconductors have shown suitable properties toward PEC applications due to appropriate conduction and valance band position and strong physical/chemical stability.^4^, ^5^ During materials selection for photoelectrode, light absorption capability and stability in electrolyte are two of the main considerations which determine the energy conversion efficiency of the PEC process.^6^ Intrinsically, n‐type metal oxides such as TiO_2_, ZnO, and SnO_2_ have been extensively investigated for photoanode in PEC cells. However, p‐type metal oxides are less studied for photocathode application. Copper oxide ideally fulfills the key requirements of photocatalyst; such as high optical absorption coefficient, earth abundant material, and can be easily processed for photocathode synthesis.^7^, ^8^, ^9^, ^10^, ^11^, ^12^, ^13^, ^14^, ^15^ Among different phases of copper oxides; cupric oxide (CuO) has relatively better photoconversion efficiency, however, it easily converts into cuprous oxide (Cu_2_O) resulting in an unstable photocathode.^7^, ^8^ Therefore, it is necessary to develop CuO‐based photocathodes which can reduce/prevent the formation of Cu_2_O phase during water‐splitting process and improve the photocorrosion stability.

Apart from stability issue, synthesis of photocathodes over large area is another challenging task to realize solar‐driven water‐splitting process is adequate for commercial production of hydrogen.^16^ Therefore, a high‐yield low cost–based electrode fabrication process is required. Sputter deposition and solution‐based thin film deposition process have been used for the synthesis of CuO.^17^, ^18^, ^19^, ^20^, ^21^ Nonvacuum‐based methods such as sol–gel deposition are suitable for thin film deposition and can reduce fabrication cost by replacing vacuum‐based deposition methods with high‐throughput and large‐scale processes. However, sol–gel‐deposited thin film has high bulk resistance, low charge transfer rate, and high recombination rate of photogenerated carriers.^22^ Fortunately, the charge transfer property can be significantly improved by incorporating carbon nanostructure in metal oxide thin films.^23^, ^24^, ^25^ Graphene‐Cu_2_O composite electrode has shown better photocatalytic performance as compared to Cu_2_O.^22^, ^23^, ^24^, ^26^ Not much attention was drawn on the development of efficient and stable graphene‐incorporated CuO photocathode. Since, CuO is a potential candidate for visible light–driven solar hydrogen production, it is necessary to develop the highly stable and efficient CuO‐based photocathode. Thus, in this work, we have specifically investigated the impact of incorporation of functionalized graphene into CuO thin film on the performance and stability of photocathode against photocorrosion. We have shown that the incorporation of –COOH‐functionalized graphene into CuO improves both the stability of photocathode and efficiency as compared to control CuO and –NH_2_‐functionalized graphene‐incorporated CuO photocathode. Because of the electron acceptor tendency of –COOH functional graphene, it captures photogenerated excess electrons and this minimizes the possibility of CuO reduction into Cu_2_O. Furthermore, by covering the CuO:G‐COOH thin film with sol–gel‐deposited TiO_2_ protecting layer, we have demonstrated highly stable all solution‐based CuO photocathode for visible light–driven solar hydrogen production.

## 2 Results and Discussion

**Figure 1:**
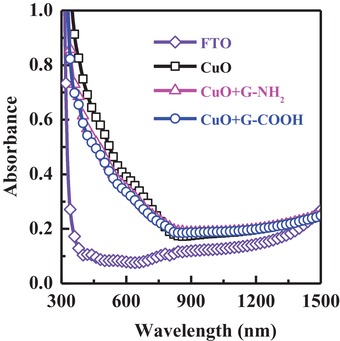
Absorbance spectra of CuO and functionalized graphene incorporated CuO films after thermal treatment at 600 °C for 10 min in furnace.

The visible light absorption properties of CuO and functionalized graphene‐incorporated CuO films are investigated before measuring the water‐splitting performance. All three electrodes (CuO, CuO:G‐NH_2_, and CuO:G‐COOH) show excellent light absorption capabilities due to the narrow bandgap of CuO. The on‐set of the visible light absorption starts at around 800 nm (**Figure** **1**) with increasing light absorption capabilities at lower wavelength. With the incorporation of functionalized graphene in CuO film, the on‐set of the light absorption spectra slightly shifts (≈−50 nm) toward lower wavelengths. There is no noticeable change in absorption profile between the two different functional groups, –NH_2_ or –COOH. As such, we can expect similar light‐harvesting properties for both films. Since these films have excellent absorption of visible light, they can potentially be good photocathodes for visible light–driven water splitting.

**Figure 2:**
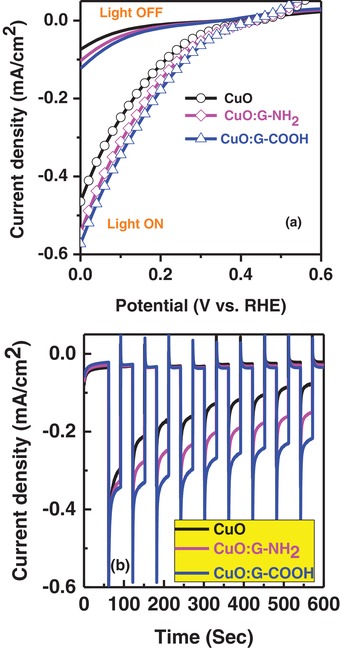
a) Current–voltage characteristics of CuO and functionalized graphene incorporated CuO (CuO:G‐NH_2_, CuO:G‐COOH) photocathode. b) Stability of CuO:G‐COOH significantly improved compared with bare CuO photocathode.

To evaluate the light‐harvesting performance of these three different electrodes (CuO, CuO:G‐NH_2_, and CuO:G‐COOH), PEC properties of CuO photocathode with and without G are tested with identical thickness of CuO and CuO:G electrodes. Linear cyclic voltammetry shows an improvement in photocurrent after G‐COOH incorporation as given in **Figure** **2**a. Current–voltage plots are recorded at a fix potential under chopped visible light conditions (Figure 2b). The dark current is relatively higher in –COOH‐functionalized graphene‐incorporated CuO electrode. It has been reported that –COOH behaves as an electron acceptor group in graphene,^27^ which can improve the p‐type conductivity of the hybrid film. It is also shown that the charge separation efficiency increases with the presence of graphene.^28^ The average photocurrent for the first excitation of visible light cycle is similar for all three electrodes, the stability of the generated photocurrent shows the importance of the functional groups. After multiple cycles of light ON‐OFF conditions, –COOH‐functionalized graphene‐incorporated CuO electrode does not show a large reduction in photocurrent. In the case of bare CuO and –NH_2_‐functionalized graphene‐incorporated CuO electrode, the reduction in photocurrent occurs rapidly and within 600 s, which generates only 20–30% of its initial photocurrent. The –COOH‐functionalized graphene‐incorporated CuO electrode rendered a stable photocurrent despite having similar light‐harvesting profile. CuO is known to be unstable under illumination and it can be easily reduced into Cu_2_O and Cu metal resulting in a decrease in photocurrent over time. As observed in Figure 2, bare CuO shows a decrease of ≈80% in photocurrent after 600 s whereas only ≈30% reduction in photocurrent is observed for –COOH‐functionalized graphene‐incorporated CuO electrode.

**Figure 3:**
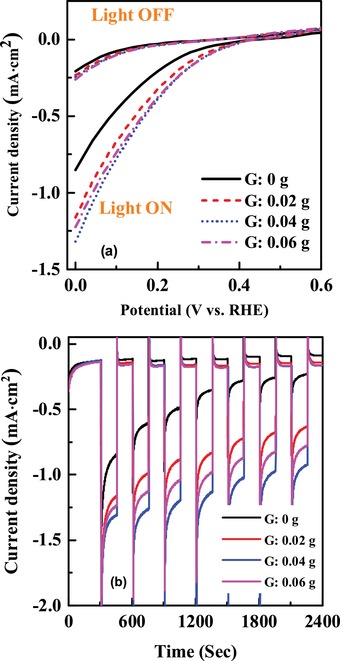
a) Current–voltage characteristics of CuO and –COOH‐functionalized graphene incorporated CuO (CuO:G‐COOH) photocathode. b) Stability of CuO:G‐COOH photocathode with different amount of graphene.

Since, –COOH‐functionalized graphene‐incorporated CuO shows promising PEC performance, we have synthesized thick CuO photocathode of ≈500 nm with different amount of –COOH‐functionalized graphene using sol–gel method as described in the Experimental Section. PEC performance varies with the thickness of photocathode. Optimum thickness for CuO‐based photocathode is ≈500 nm.^12^, ^17^ The –COOH‐functionalized graphene of 0.02, 0.04, and 0.06 g added separately into 1 mL of copper oxide solution to synthesize CuO:G‐COOH with different amount of graphene. Linear cyclic voltammetry in **Figure** **3**a shows variation of photocurrent with different amount of G‐COOH incorporation. The CuO:G‐COOH photocathode with 0.02 g produces a photocurrent density of −1.16 mA cm^−2^ at 0 V versus reversible hydrogen electrode (RHE), and the photocurrent density is increased up to −1.32 mA cm^−2^ when the amount of –COOH‐functionalized graphene is increased to 0.04 g. The photocurrent density of CuO:G‐COOH photocathode with 0.06 g is lower than the CuO:G‐COOH photocathode with 0.04 g. Initial improvement of photocurrent by increasing the amount of –COOH‐functionalized graphene is due to the enhancement of conductivity. While further increasing the amount of –COOH‐functionalized graphene increases the recombination and degrades the performance of CuO:G‐COOH photocathode. The stability of the photocathode also significantly depends on the amount of G‐COOH, as shown in Figure 3b. In the case of control CuO electrode, the reduction in photocurrent occurs rapidly and within 2400 s, it generates only 20–30% of its initial photocurrent, whereas, ≈30% reduction in photocurrent is observed for best‐performing –COOH‐functionalized graphene‐incorporated CuO electrode. Thus, CuO:G‐COOH photocathode with optimized concentration can be a suitable for solar hydrogen generation, as it enhanced the photogenerated carriers and charge transport properties of CuO.

**Figure 4:**
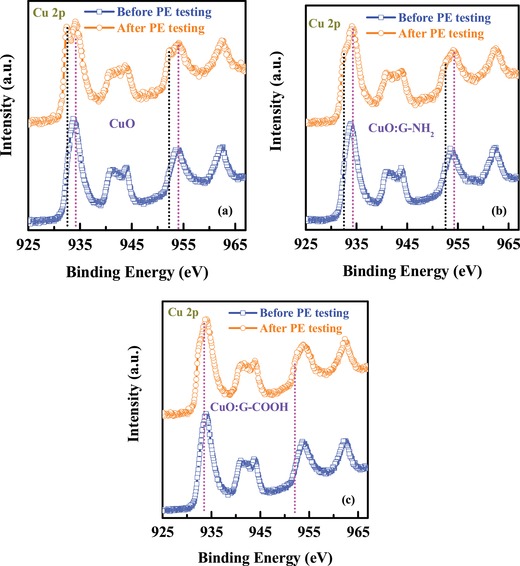
XPS spectra of Cu 2p core level spectra from a) CuO photocathode, b) CuO:G‐NH_2_ photocathode, and c) CuO:G‐COOH photocathode.

To understand the improvement in performance of CuO:G‐COOH film compared with control CuO and –NH_2_‐functionalized graphene‐incorporated CuO, X‐ray photoelectron spectroscopy (XPS), X‐ray diffraction (XRD), Raman characteristics, and impedance spectroscopic analysis were conducted. A VG ESCALAB 220i‐XL XPS system with a monochromatic Al Kα source (1486.6 eV) was used to estimate the surface chemical composition of thin films. XPS spectra of the prepared samples are measured before and after PEC tests. **Figure** **4** shows the Cu 2p XPS spectra of the CuO, CuO:G‐NH_2_, and CuO:G‐COOH thin films. Satellite peaks in the range of 940–950 and 958–970 eV are observed. It also shows strong Cu 2p_3/2_ peak at 933.8 eV and Cu 2p_1/2_ peak at 953.8 eV for all film which confirms the formation of CuO dominant phase.^17^, ^29^ However, after PEC measurements, small shoulder peaks at 932.3 and 952.3 eV are observed for CuO and CuO:G‐NH_2_ films. On the other hand for CuO:G‐COOH thin film, the intensity of small shoulder peak at 932.3 and 952.3 eV is much lower compared with bare CuO and CuO:G‐NH_2_ thin films. These observations indicate the reduction of CuO to Cu_2_O is quite prominent for CuO and CuO:G‐NH_2_ photocathodes than CuO:G‐COOH photocathode.

**Figure 5:**
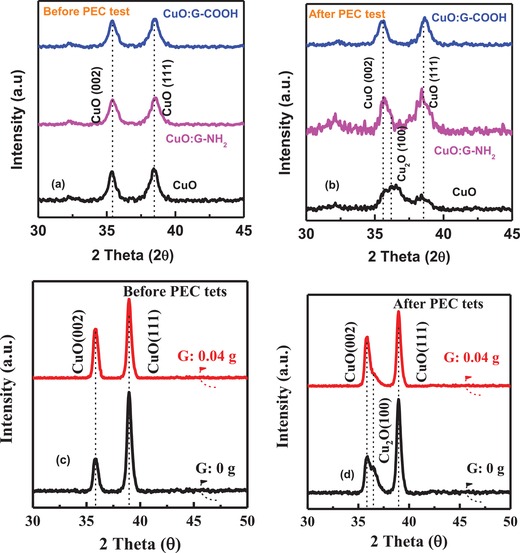
XRD spectra of CuO (100 nm), CuO:G‐NH_2_ (100 nm), and CuO:G‐COOH (100 nm) photocathode a) before PEC test and b) after PEC test. For CuO and CuOG‐NH_2_, mixed phases of CuO and Cu_2_O observed after PEC test. XRD spectra of CuO (500 nm) and CuO:G‐COOH (500 nm) photocathode c) before PEC test and d) after PEC test.

To evaluate the structural property of the CuO photocathode before and after PEC experiment, XRD analysis has been performed. XRD spectra of thin film CuO and G‐incorporated CuO were presented in **Figure** **5**a. As shown in this figure, before PEC test, bare CuO and G‐incorporated CuO films have two distinct peaks at 35.4° and 38.75° which corresponds to the (002) and (111) plane for CuO, respectively (JCPDS# 05–0661). However, after PEC test, there is XRD peak at 36.4° for bare CuO and CuO:G‐NH_2_, which is ascribed to the Cu_2_O (111) phase (JCPDS# 05–0667) (Figure 5b). The phase transition is more prominent for thick CuO and CuO:G‐COOH photocathodes of thickness ≈500 nm before and after PEC test, as shown in Figure 5c,d, respectively. By incorporating –COOH‐functionalized graphene with CuO, phase transformation during PEC can be significantly reduced and thus enhanced photocorrosion stability of the CuO photocathode.

**Figure 6:**
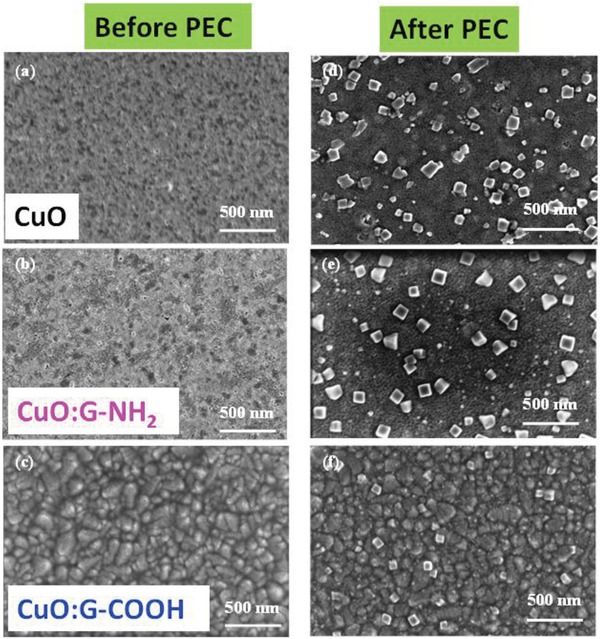
Surface morphology of spin‐coated a) CuO, b) CuO:G‐NH_2_, and c) CuO:G‐COOH thin films before photocurrent measurement, respectively. Surface morphology of d) CuO, e) CuO:G‐NH_2_, and f) CuO:G‐COOH thin films after photocurrent measurement, respectively. The formation of Cu_2_O significantly reduced for CuO:G‐COOH photocathode.

The reduction of CuO into Cu_2_O during water‐splitting process is also identified in scanning electron microscopic (SEM) imaging. **Figure** **6** shows the SEM images of CuO and functional graphene (–NH_2_ and –COOH) incorporated CuO cathodes before and after water‐splitting measurements. It is worth to note that the formation of Cu_2_O during water splitting is responsible for the poor stability of CuO photocathode. Figure 6 shows top‐surface of the films after water‐splitting testing. The cubicle structures on the surface reveal the formation of Cu_2_O during charge transfer.^30^, ^31^, ^32^, ^33^ The density of nanostructures is very high for bare CuO and CuO:G functionalized with –NH_2_. However, for CuO:G functionalized with –COOH, the size and density of the cubicle structures are considerably reduced. Thus, SEM analysis also supports the XPS observation of Cu_2_O phase formation after PEC measurements. Thus, we can say formation of unwanted Cu_2_O can be reduced significantly through the incorporation of functionalized graphene into the CuO film.

**Figure 7:**
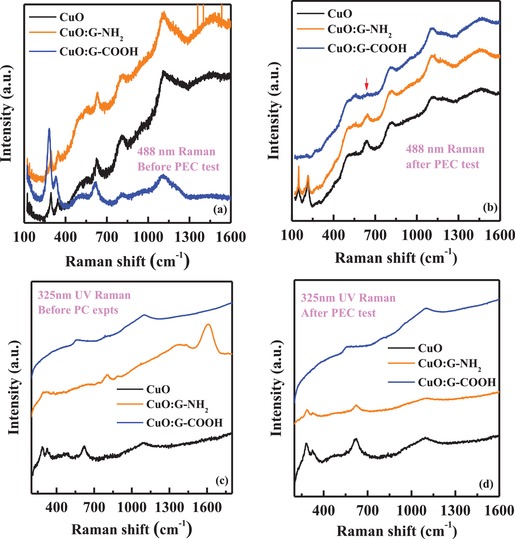
Raman spectra recorded from the samples before and after PEC studies. a,b) Spectra with 488 nm visible Raman excitation. c,d) Spectra with 325 nm UV Raman excitation from three sets of samples (CuO, CuO:G‐NH_2_, and CuO:G‐COOH).

In order to address the vibrational properties of these CuO‐based photocathodes with and without incorporation of functionalized graphene, Raman spectroscopy measurements were performed. **Figure** **7** shows the Raman spectra of the samples before and after PEC tests. The 488 nm Raman excitations (Figures 7a,b) probe the whole film while the 325 nm UV excitation (Figures 7c,d) probes the chemical bonding at the surface. Raman peaks from the CuO‐based samples are superimposed on the background substrate fluorescence bands with visible Raman excitation, with signature peaks from both CuO‐ and Cu_2_O‐based complexes dominate in the range of 200–700 cm^−1^. The laser excitation power was kept very low to avoid laser‐induced heating or degradation of surface. The visible Raman spectrum in Figure 7a shows prominent phonon peaks at 295, 344, and 628 cm^−1^ from CuO film before PEC tests. The crystalline CuO shows three Raman active modes (A_g_ + 2B_g_), with A_g_ mode around 297 cm^−1^ and 2B_g_ modes around 344 and 629 cm^−1^.^34^ After PEC tests, the visible Raman spectrum from the same CuO sample shows significant changes of vibrational modes, with prominent peaks appearing at 148, 216, and 640 cm^−1^. The Raman active phonon modes of Cu_2_O usually appear around 148–158 cm^−1^ (Γ_15_^−1^: LO), 285 cm^−1^ (2Γ_15_^−1^: LO), and 620 cm^−1^ (Γ_15_^−2^: TO), while modes around 216–218 cm^−1^ represent second‐order Raman mode of Cu_2_O crystal.^35^ The disorder‐induced crystallization in these films also leads to the observation of a Raman peak in the 635–645 cm^−1^ range as an IR allowed mode.^36^, ^37^ Thus, visible Raman clearly shows the PEC‐induced respective changes toward Cu_2_O reduction phase in our experiments with starting CuO film. With the addition of graphene complexes, the Raman modes from the films show detectable peak shifts and broadening. Visible Raman spectrum from the CuO:G‐NH_2_ film shows similar modes at 296, 348, and 631 cm^−1^ before PEC tests, and after PEC tests the peaks appear at 149, 217, and 644 cm^−1^ indicating clear reduction to Cu_2_O phase. Broader modes around 810 and 1100 cm^−1^ originate from the multi‐phonon processes with contribution from the wagging mode of C—H and the sp^3^ bonding of carbon complexes. The CuO and CuO:G‐NH_2_ films show similar Raman spectra before and after PEC test. On the other hand, CuO:G‐COOH hybrid film shows remarkable changes in the phonon spectra before and after PEC tests. For the case of CuO:G‐COOH, Raman modes at 283, 330, and 618 cm^−1^ appeared prior to PEC tests due to changes in the chemical bonding with graphene. The nature of the bonding gave rise to presence of LO and TO phonons of Cu_2_O phase within the film gradient while peak at 327–330 cm^−1^ is related to disordered activated mode arising from the CuO phase chemical bonding. It is noteworthy to see that after PEC tests, the mode at 644 cm^−1^ is absent while no prominent modes of Cu_2_O phase is seen in the spectra recorded from CuO:G‐COOH. This observation supports the stable performance of the CuO:G‐COOH films as seen from our water‐splitting experiments.

To further address the changes on the chemical bonding at the surface, the UV Raman excitation was used due to very shallow penetration depth. The Raman spectra in Figure 7c show prominent combination modes at 291, 327, and 622 cm^−1^ from the top surface of the CuO film, and these modes appear at the expense of pure CuO phonon modes as seen from visible Raman. After PEC tests, the same sample shows spectral line shape changes with modes appearing at 280, 326, and 622 cm^−1^. The intense mode at 622 cm^−1^ after PEC tests suggests contribution of TO–phonon vibration mode of Cu_2_O phase at the surface similar to the case of visible Raman data. The CuO:G‐NH_2_ film under UV Raman excitation shows weaker peaks at 296 cm^−1^ while modes related to graphene bonding related to CuO*_x_* can be seen at 804, 1380 (D‐band), and 1611 cm^−1^ (G‐band). After PEC tests, all these modes became much weaker but prominent peaks at 287, 326, and 623 cm^−1^ dominate the UV Raman spectrum from CuO:G‐NH_2_. The appearance of these peaks shows the reduction to Cu_2_O phase with the presence of disorder at the surface. However, in the case of UV Raman measurements from the surface of the CuO:G‐COOH film, broad peaks at 567 and 1100 cm^−1^ are seen prior to PEC tests. After PEC experiment, similar modes appear where sp^3^ bonding of carbon on the surface still dominates in the form of Raman peak at 1100 cm^−1^. These observations also confirm the absence of Cu_2_O reduction at the surface of the CuO:G‐COOH film after PEC tests and led to stable performances of photocatalytic activity.

The remarkable change in stability with the hybrid electrode (CuO:G‐COOH) might be related to the following causes. Graphene incorporation induces faster transportation of photogenerated charge carrier to their respective electrodes resulting in efficient separation of large numbers of long‐lived e^−^/h^+^ pairs.^28^ In addition, because of the electron accepting nature of –COOH functional group in graphene, the photogenerated electrons can be easily captured which results in reducing the availability of free electrons for conversion of CuO into Cu_2_O leading to a more stable photocurrent.^27^ For –NH_2_‐functionalized graphene‐incorporated CuO electrode (CuO: G‐NH_2_), the –NH_2_ functional group works as electron donor in graphene.^27^ Due to the electron donor nature of –NH_2_, excess electrons are supplied to CuO and reduce it into Cu_2_O. This negates the beneficial effects of graphene resulting in an unstable photocurrent.

**Figure 8:**
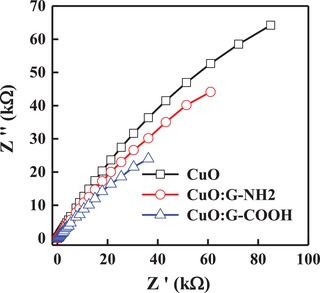
Nyquist plot of CuO, CuO:G‐NH_2_, and CuO:G‐COOH photocathode.

The charge transfer resistance (*R*_ct_) significantly decreases from a value of 101 kΩ cm^2^ (CuO photocathode) to 28 kΩ cm^2^ after incorporation of G‐COOH into CuO film, as shown in Nyquist plot (**Figure** **8**). The reduction in *R*_ct_ value shows an increase in charge transfer activity for the CuO:G‐COOH composite film, which is due to the presence of electron acceptor group. With the increase in charge transfer activity, there is a decrease in recombination of photogenerated charges leading to an improvement in the PEC performance. Performance of the CuO:G‐COOH photocathode is comparable with sputter grown CuO‐based photocathode.^38^ For the development of efficient CuO‐based visible light–driven photocathode, incorporation of functionalized graphene is essential to enhance the charge transport property of the CuO thin film.

**Figure 9:**
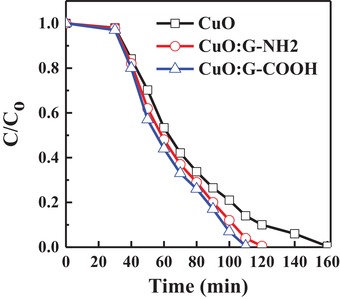
Comparison of MB. C/Co versus time of CuO, CuO:G‐NH_2_, and CuO:G‐COOH.

The graphene‐incorporated CuO photocathodes (CuO, CuO:G‐NH_2_, and CuO:G‐COOH) also investigated for degradation of methylene blue (MB) under sun‐light illumination. Concentration versus time plots for photocatalytic degradation of MB are shown in **Figure** **9**. A control experiment was carried out to show that photodegradation is not apparent without the using of a photocatalyst. The MB concentration is significantly reduced by adding G‐NH_2_ and G‐COOH. Among the samples used for MB degradation, CuO:G‐COOH shows the best performance for the photocatalytic degradation of MB under sun‐light illumination, highlighting the key role of unique structure of CuO:G‐COOH in MB photocatalytic degradation. The G‐incorporated CuO is not only enhanced photocorrosion stability, it also exhibits faster MB degradation compared with CuO photocathode. Performance of CuO‐based photocathode for MB degradation is shown in **Table** **1**.

**Table 1:** Comparison of CuO‐based photocathode for MB degradation

Photocathode material	Dimension/Thickness	Method	Dye degradation time	Refs.
Poly‐*o*‐phenylenediamine‐modified TiO_2_	30–50 nm	Photocatalysis (1000 W xenon lamp)	≈180 min	39
Stoichiometric CuO and Cu_2_O	500 nm	Photocatalysis (300 W xenon lamp)	≈225 min	40
TiO_2_/active carbon	20 nm	Photocatalysis (15 W UV bench lamp)	≈120 min	41
ZnO	73 nm	UV lamp irradiation	≈120 min	42
Cu*_x_*O‐ZnO composite	20 nm	i) Photocatalytically cleaning the ZnO materials (24 W) ii) Simulated solar light for MB degradation at 30 W m^−2^	≈90 min	43
Nanoporous SnO_2_−ZnO	27 nm	Photocatalysis (125 W high pressure mercury lamp)	≈80 min	44
Au‐decorated SnO_2_	25–30 nm	Visible light	≈240 min	45
TiO_2_/Ag/SnO_2_ composites	≈30 nm	Photocatalysis (500 W xenon lamp)	≈140 min	46
Pd nanostructures into thin CuO film	200 nm	Photocatalysis (150 W xenon arc lamp)	≈90 min	47
TiO_2_ powder	Not available	Photocatalysis (150 W xenon arc lamp)	≈110 nm	48
Thin CuO film	Not available	Photocatalysis (150 W xenon arc lamp)	≈150 min	49
Faceted TiO_2_	Not available	Photocatalysis (150 W xenon arc lamp)	≈30 min	50
CuO:G‐COOH	100 nm	Photocatalysis (150 W xenon arc lamp)	≈100 min	This work

**Figure 10:**
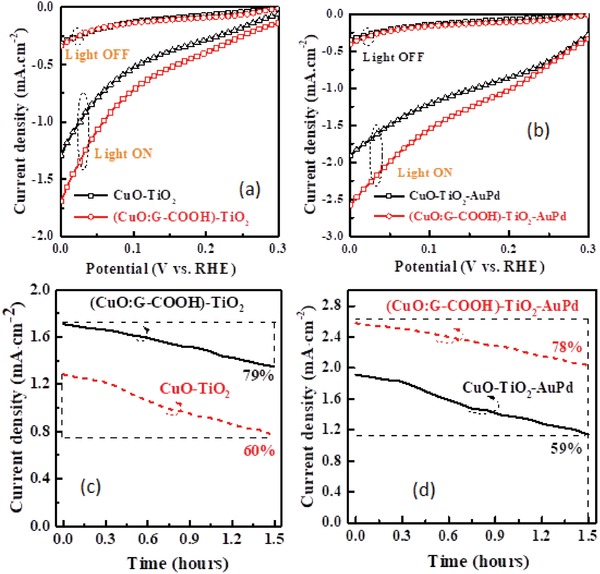
Current–voltage characteristics of CuO and CuO:G‐COOH photocathode with a) TiO_2_ surface protecting layer and b) TiO_2_‐Au‐Pd nanostructure. Photocorrosion stability of c) CuO‐TiO_2_ and (CuO:G‐COOH)‐TiO_2_ photocathodes and d) CuO‐TiO_2_ and (CuO:G‐COOH)‐TiO_2_ photocathodes with Au‐Pd decoration.

**Figure 11:**
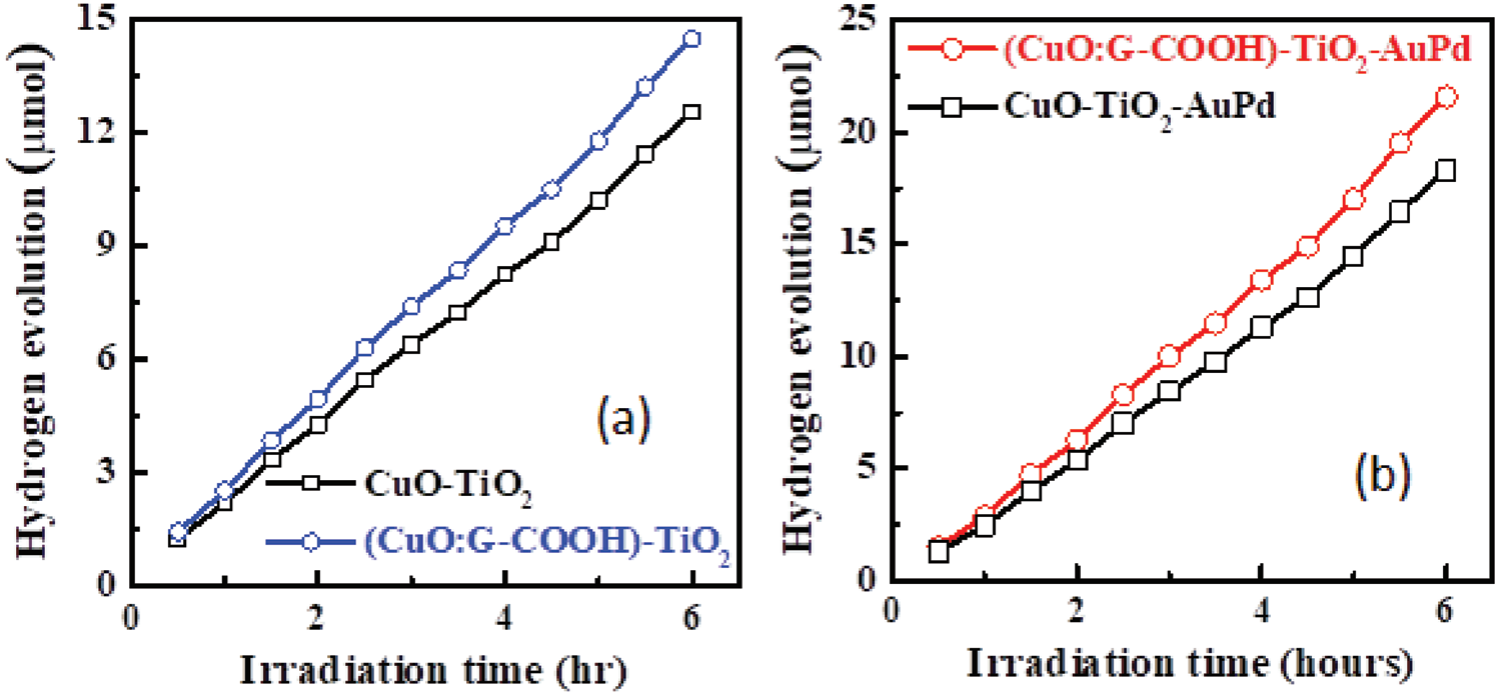
Hydrogen evolution of CuO and (CuO:G‐COOH) photocathodes with a) TiO_2_ passivation and b) TiO_2_‐Au‐Pd nanostructure under standard light illumination.

CuO:G‐COOH thin film integrated with sol–gel‐derived TiO_2_ protecting layer and Au‐Pd co‐catalyst nanostructures to enhance the stability and performance of the photocathode further. The TiO_2_ protecting layer of 50 nm was deposited on top of control CuO and CuO:G‐COOH photocathodes. The Au‐Pd nanostructures were decorated on the surface through sputtering of Au‐Pd (60:40) target using a JEOL smart coater. **Figure** **10** shows the PEC current–voltage characteristics and photocurrent stability of the prepared photocathodes with TiO_2_ protecting layer and decorated with Au‐Pd nanostructures. The photocurrent enhances for both CuO:TiO_2_ CuO:G‐COOH:TiO_2_ with Au‐Pd nanostructures. The long‐term photocorrosion stability of (CuO:G‐COOH)‐TiO_2_‐AuPd photocathode increased as compared to the CuO‐TiO_2_‐AuPd photocathode. Hydrogen evolution of (CuO:G‐COOH)‐TiO_2_‐Au‐Pd and CuO‐TiO_2_‐AuPd photocathodes under light illumination is presented in **Figure** **11**. The CuO:G‐COOH photocathode with TiO_2_ surface passivation and Au‐Pd nanostructure shows 28% higher amount of hydrogen evolution compared to the CuO‐TiO_2_‐Au‐Pd photocathode. Photocatalytic performance and hydrogen evolution using CuO‐based photocathode is shown in **Tables** **2** and **3**. For solar‐driven hydrogen generation, it is essential to develop highly stable and low‐cost photocathode. The present study shows that the functionalized graphene‐incorporated CuO photocathode can be synthesized through solution technique and it is stable. Thus, it can open up the opportunity to develop solar hydrogen generation for large scale.

**Table 2:** Comparison of photocurrents for CuO‐based electrode

Photocathode material	Synthesis process	Photocurrent	Thickness	Refs.
CuO thin film	Sputter	−0.65 mA cm^−2^ @ −0.45 V (Ag/AgCl)	≈500 nm	12
NiO‐CuO thin film	Sol–gel	−1.07 mA cm^−2^ @ −0.55 V (Ag/AgCl)	850 nm	17
CuO thin film	RF‐magnetron sputtering	−3.1 mA cm^−2^ @ 0 V (RHE)	500 nm	38
Au‐Pd‐decorated CuO thin film	RF‐magnetron sputtering	−3.88 mA cm^−2^ @ 0 V (RHE)	500 nm	38
CuO thin film	Sol−gel process	−0.55 mA cm^−2^ @ 0.05 V (RHE)	600 nm	51
CuO thin film	Electrolysis deposition‐thermal oxidation	−1.8 mA cm^−2^ @ 0 V (RHE)	not provided	52
Li‐doped CuO nanoparticles	Flame spray pyrolysis	−1.7 mA cm^−2^ @ −0.55 V (Ag/AgCl)	1700 nm	53
CuO nanoparticles films	Solution process	−1.2 mA cm^−2^ @ −0.55 V (Ag/AgCl)	1340 nm	54
CuO thin film	Sol–gel method	−0.7 mA cm^−2^ at 0 V versus RHE	≈500 nm	This work
CuO thin film with TiO_2_ protecting layer (CuO‐TiO_2_)	Sol–gel method	−1.3 mA cm^−2^ at 0 V versus RHE	≈500 nm	This work
CuO thin film with TiO_2_ protecting layer and Au‐Pd co‐catalyst nanostructures (CuO‐TiO_2_‐AuPd)	Sol–gel method and RF sputtering	−1.9 mA cm^−2^ at 0 V versus RHE	≈500 nm	This work
–COOH‐functionalized graphene into the CuO film (CuO:G‐COOH)	Sol–gel method	−1.32 mA cm^−2^ at 0 V versus RHE	≈500 nm	This work
–COOH‐functionalized graphene into the CuO film (CuO:G‐COOH) with TiO_2_ protecting layer (CuO:G‐COOH)‐TiO_2_	Sol–gel method	−1.75 mA cm^−2^ at 0 V versus RHE	≈500 nm	This work
–COOH‐functionalized graphene into the CuO film (CuO:G‐COOH) with TiO_2_ protecting layer and Au‐Pd co‐catalyst nanostructures (CuO:G‐COOH)‐TiO_2_‐AuPd	Sol–gel method and RF sputtering	−2.5 mA cm^−2^ at 0 V versus RHE	≈500 nm	This Work

**Table 3:** Comparison of hydrogen evolution using CuO‐based photocathode

Type of photocathode	Fabrication process	Hydrogen evolution reaction	Thickness of CuO	Refs.
CuO thin film	RF‐magnetron sputtering	≈2 µmol h^−1^	500 nm	38
Au‐Pd‐decorated CuO thin film	RF‐magnetron sputtering	≈2.6 µmol h^−1^	500 nm	38
CuO thin film with TiO_2_ protecting layer (CuO‐TiO_2_)	Sol–gel method	≈2.1 µmol h^−1^	≈500 nm	This work
CuO thin film with TiO_2_ protecting layer and Au‐Pd co‐catalyst nanostructures (CuO‐TiO_2_‐AuPd)	Sol–gel method and RF sputtering	≈2.5 µmol h^−1^	≈500 nm	This work
–COOH‐functionalized graphene into the CuO film with TiO_2_ protecting layer (CuO:G‐COOH)‐TiO_2_	Sol–gel method	≈2.3 µmol h^−1^	≈500 nm	This work
–COOH‐functionalized graphene into the CuO film with TiO_2_ protecting layer and Au‐Pd co‐catalyst nanostructures (CuO:G‐COOH)‐TiO_2_‐AuPd	Sol–gel method and RF sputtering	≈3.0 µmol h^−1^	≈500 nm	This work

## 3 Conclusions

A simple, solution‐based approach was presented to design and fabricate functionalized graphene‐incorporated CuO photocathodes. The –COOH functional group has shown advancement in water‐splitting performance with improvement in the stability of the CuO electrodes. Through incorporation of functionalized graphene, the CuO‐based films can be utilized to realize the solar‐driven water splitting for hydrogen production and photocatalytic activity. In addition, it is observed that type of functionality on graphene played an important role in making a more stable and efficient photoelectrode. These observations are also supported by spectroscopic measurements. Functional groups with electron accepting tendency are thus beneficial in improving the stability of p‐type CuO photocathodes. Furthermore, by integrating surface passivation layer stability of the CuO:G‐COOH can be pushed further. The CuO:G‐COOH photocathode with TiO_2_ surface passivation shows hydrogen evolution rate of ≈2.3 µmol h^−1^. The sol–gel‐developed CuO with functionalized graphene and surface protecting layer shows promising characteristics for visible light–driven solar hydrogen production. This work opens up the development of highly stable and efficient photocathode through solution technique and by combining the metal oxides for visible light–driven solar hydrogen production.

## 4 Experimental Section

Thin film of CuO was deposited by spin coating CuO sol solution onto fluorine‐doped tin oxide (FTO) glass substrates.^17^ The copper oxide sol was prepared by adding 1.0 mL ethanolamine to 20 mL of 2‐methoxyethanol under vigorous stirring. After which, 1.09 g of copper (II) acetate was added into the solution and a deep blue solution was formed after 15 min of continuous stirring. Then, 0.5 mL of polyethylene glycol (average molecular weight of 200) was added into the solution and the solution was ready to be used to deposit CuO film. To incorporate graphene into copper oxide film, 0.04 g of –COOH‐functionalized graphene nanosheets and –NH_2_‐functionalized graphene nanosheets (Cheap Tubes Inc., USA) were added separately into 1 mL of copper oxide solution. The solution was then sonicated with a probe using pulse mode (5 s on and 5 s off) for 1 min. This was followed by an additional stirring for 3 h before deposition onto FTO glass substrate through spin‐coating method. Then film was dried on a hotplate at 100 °C for 2 min before annealing at 600 °C for 10 min. The thickness of CuO and CuO‐G films was ≈100 nm, measured by using surface profilometer.

PEC measurement was carried out in a three‐electrode cell with cupric oxide films as working electrode, platinum foil as counter electrode, and Ag/AgCl for the reference electrode. The measurements were performed in 0.1 m Na_2_SO_4_ aqueous electrolyte solution (pH 5.84). An active area of 1 cm^2^ was defined on the copper oxide film by covering the films with black tape. Metrohm‐Autolab PGSTAT101 potentiostat was used for PEC characterization with a 150 W Xenon arc lamp fitted with AM 1.5 filters as the light source. The intensity of the light illuminated on the copper oxide samples was maintained at 100 mW cm^−2^. To determine charge transfer resistance, EIS was performed within frequency range of 10^5^–10^−2^ Hz at an open circuit potential and 10 mV amplitude. The micro‐Raman spectroscopy measurements were performed on all the samples before and after PEC characterization. In order to address the nature of phase changes at the surface and substrate interface, the samples were excited with 325 nm (UV) and 488 nm (visible) Raman excitation wavelengths using a JY‐LABRAM system.

Graphene‐incorporated CuO (CuO, CuO:G‐NH_2_, and CuO:G‐COOH) photocathodes were used for MB photocatalytic degradation. The samples were placed at the bottom of a beaker and 20 mL of 10.0 mg L^−1^ MB solution was used to carry out the experiments. The beaker was placed on a shaker (50 rpm, ambient temperature) for 30 min in the dark. For the photocatalytic experiments, a 150 W xenon arc lamp equipped with an air mass 1.5 global (AM 1.5G) was used as the light source. After photocatalytic degradation of the required duration, 2 mL from each experiment was sampled and their concentrations were analyzed. Equation (1) was used to determine the MB photocatalytic degradation rate (%) in aqueous solution

(1) $$\text{Photocatalytic}\;\text{degradation}\;\text{rate}\;(\%)\;\;\text{=}\;\;\frac{C_{0}-C_{\text{t}}}{C_{0}}\times 100$$

## Conflict of Interest

The authors declare no conflict of interest.
